# Breakdown of category-specific word representations in a brain-constrained neurocomputational model of semantic dementia

**DOI:** 10.1038/s41598-023-41922-8

**Published:** 2023-11-10

**Authors:** Yury Shtyrov, Aleksei Efremov, Anastasia Kuptsova, Thomas Wennekers, Boris Gutkin, Max Garagnani

**Affiliations:** 1 Center of Functionally Integrative Neuroscience (CFIN), Institute for Clinical Medicine, Aarhus University, Aarhus, Denmark; 2 Centre for Cognition and Decision Making, Institute for Cognitive Neuroscience, HSE University, Moscow, Russia; 3 Montreal Neurological Institute-Hospital, McGill University, Montreal, Quebec Canada; 4 School of Engineering, Computing and Mathematics, University of Plymouth, Plymouth, UK; 5 Département d’Etudes Cognitives, École Normale Supérieure, Paris, France; 6 Department of Computing, Goldsmiths - University of London, London, UK; 7 Brain Language Laboratory, Department of Philosophy and Humanities, Freie Universität Berlin, Berlin, Germany

**Keywords:** Human behaviour, Cognitive neuroscience, Computational neuroscience, Diseases of the nervous system, Learning and memory, Neural circuits, Neurology

## Abstract

The neurobiological nature of semantic knowledge, i.e., the encoding and storage of conceptual information in the human brain, remains a poorly understood and hotly debated subject. Clinical data on semantic deficits and neuroimaging evidence from healthy individuals have suggested multiple cortical regions to be involved in the processing of meaning. These include semantic hubs (most notably, anterior temporal lobe, ATL) that take part in semantic processing in general as well as sensorimotor areas that process specific aspects/categories according to their modality. Biologically inspired neurocomputational models can help elucidate the exact roles of these regions in the functioning of the semantic system and, importantly, in its breakdown in neurological deficits. We used a neuroanatomically constrained computational model of frontotemporal cortices implicated in word acquisition and processing, and adapted it to simulate and explain the effects of semantic dementia (SD) on word processing abilities. SD is a devastating, yet insufficiently understood progressive neurodegenerative disease, characterised by semantic knowledge deterioration that is hypothesised to be specifically related to neural damage in the ATL. The behaviour of our brain-based model is in full accordance with clinical data—namely, word comprehension performance decreases as SD lesions in ATL progress, whereas word repetition abilities remain less affected. Furthermore, our model makes predictions about lesion- and category-specific effects of SD: our simulation results indicate that word processing should be more impaired for object- than for action-related words, and that degradation of white matter should produce more severe consequences than the same proportion of grey matter decay. In sum, the present results provide a neuromechanistic explanatory account of cortical-level language impairments observed during the onset and progress of semantic dementia.

## Introduction

Semantic knowledge can be defined as the information about the meaning of concepts and words accumulated by an individual through their experience^1,2^. This knowledge is paramount for our daily lives: we use it continuously to understand our physical environment and interact with it, to communicate our thoughts to others as well as to comprehend messages addressed to us. Difficulties experienced by people who suffer from semantic memory impairments are manifest, for example, in deficits in the ability to recognise or make use of common objects^3,4^ and in impairments of language comprehension and production^5,6^. In spite of its obvious importance and the devastating effects of any impairments, the neurobiological mechanisms underpinning the brain’s semantic system—i.e., the encoding and storage of meaning—are still poorly understood and remain the object of an ongoing debate^7–9^. One influential view on semantic processing is offered by theories of embodied/grounded cognition, according to which concepts are grounded in perception and action, so that their processing—e.g., during language comprehension—heavily relies upon the brain’s modality-specific sensory and motor systems^9–13^. Although this framework is supported by a growing body of experimental evidence^1,14–18^, this evidence is not entirely undisputed^19–21^. For instance, some scholars claim that sensorimotor systems play only a secondary role in conceptual and language processing^22,23^. Others highlight that embodied semantics theories may be difficult to reconcile with our ability to acquire and process abstract concepts^24,25^, although, in the domain of language, some proposals addressing this latter aspect have also been offered^9,26–28^.

Important evidence on the functioning of the semantic system comes from clinical studies. Of particular interest is the so-called semantic dementia (SD), a neurodegenerative disorder characterised by a progressive deterioration of semantic knowledge across different conceptual categories; in the language domain, core symptoms include anomia and single-word comprehension impairments^5,6,29^. Whereas the term “semantic dementia” focuses on the language-specific features of this condition, it arises as a result of degradation in fronto-temporal cortices (frontotemporal lobar degeneration, FTLD^30^) and is sometimes termed to be a variant of fronto-temporal dementia (FTD). Similar neuropathological/neurodegenerative patterns are known to underpin primary progressive aphasia (PPA), a disease characterised by a progressive loss of the language function, with other cognitive functions relatively preserved^31^. Its semantic variant (svPPA), presenting with fluent speech but impaired word comprehension, is often equated to SD, with SD patients fulfilling criteria for PPA^32^, although there is no full agreement in the literature in that respect^33^. This paper uses the term “semantic dementia” (without any specific intention to differentiate it from svPPA), which highlights the semantically-specific nature of the disorder.

Neurologically, the key feature distinguishing SD patients from other neurodegenerative diseases is the gradual atrophy of the anterior temporal lobes (ATL), which is usually left-lateralised^34–38^ and accompanied by a progressive loss of semantic knowledge (agnosia) and word comprehension abilities. The study of SD patients^5,35,39,40^ has enabled important insights into conceptual information processing mechanisms in the brain such as the hierarchy of features in lexico-semantic representations.

An influential theoretical framework which explains various aspects of the semantic memory system and SD data is the so-called “hub-and-spokes” model^41–43^. It proposes that a higher-order amodal convergence area (“hub”), located in anterior temporal regions, acts as a processing centre and a connector between different modality-specific cortices (“spokes”), which, in turn, store the concepts’ specific referential (e.g., sensory, motor) features. This theory has been successful in explaining a large variety of behavioural and neuroimaging results on semantic processing in healthy controls and clinical populations; yet, the exact mechanisms underpinning the functioning of the semantic system and its breakdown in SD remain poorly understood. An increasingly important and productive avenue for tackling the mechanistic principles of the human neurocognitive operations is the use of neurocomputational models, which, unlike diverse clinical populations, offer a controlled environment for manipulating different properties in specific processing systems. Indeed, a number of computational implementations have been successful in modelling various aspects of the semantic system and its deficits, SD included^40,44–51^. One important assumption common to these computational accounts is that the meaning of words and symbols is localised to the ATL “hub” area. In such simulation studies, this is normally the rationale behind presenting “meaning patterns” to the model correlate of the ATL during the network training, as a way to mimic concept acquisition. Typically, after lesioning different parts of the model, reconstruction/recollection of such learnt meaning patterns is degraded (to different extents). The conclusion normally drawn from this observation is that the (simulated) lesions induce a semantic/comprehension “impairment” in the model^40,44,49–51^. This conclusion, however, seems warranted entirely by the key initial assumption, i.e., that there is a “module” responsible for processing meaning, and that such a module is located in the ATL. In short, the somewhat circular logic on which these studies implicitly rely goes roughly as follows: (1) the model correlate of the semantic system is located in the ATL; (2) as a result of lesions to the ATL (and/or other model parts), activity in ATL is degraded; therefore, (3) the model’s semantic system is impaired. However, if the key hypothesis (1) does not hold—for example, if, as claimed by the embodied cognition theories, the brain’s concepts are not stored and processed locally, but are instead represented in a *distributed* manner, across a number of cortical areas—simply looking at the activity in ATL (or in any other specific area) alone would be insufficient to assess the model’s comprehension abilities.

Furthermore, although the most recent computational implementations of the hub-and-spokes framework do map model areas and links between them to corresponding brain areas and neuroanatomical connections, such models still include a number of aspects which may not be neurobiologically plausible. For example, in those cases where learning is modelled, synaptic plasticity is simulated using a gradient-dependent mechanism, known as back-propagation^52^. This learning rule relies on the presence of a global “error” signal, computed as the difference between the network’s expected (target) and measured (actual) response in a pre-specified set of (“output”) units. Whilst very effective from an engineering point of view (for a review, see^53^), the issue of whether gradient-dependent learning is actually happening in the human brain remains controversial^54–56^. Although recent works^57,58^ have hypothesised that “top-down” feedback projections in the sensory processing hierarchy—normally believed to mediate attentional information^59^—could, in principle, carry such a global error signal, they also acknowledge that the role these anatomical connections play in cortical computations remains unclear, and agree that, to date, one “cannot say that the cortex employs backprop-like learning”^57^. Other non-neurobiologically grounded aspects typically include “all-to-all” between-area links (i.e., each artificial cell of an area projects to all cells in the next area), known not to be feasible in the brain^60^, mono-directional between-area links (if existing, between-area connections tend to be mostly reciprocal in the brain^61,62^), and the lack of recurrent within-area links and of inhibitory cells or inhibitory mechanisms, both of which are in fact pervasive in the brain^60,63^. In sum, whereas such models have been hugely important in advancing neurocomputational investigations of the human brain in general and semantic systems/deficits in particular, they can still be improved upon by incorporating additional constraints necessary for a *neurobiologically realistic* modelling of human cognitive functions^56^.

Here, we adopt a biologically grounded approach to modelling cortical semantic processing and use a brain-realistic neural architecture to simulate the natural emergence of semantic structures in a bottom-up way, without a priori imposing a specific cortical locus for concepts and meaning on the model or on the brain. By “natural” and “bottom-up” we mean here that the model autonomously learns, in a completely unsupervised manner, conceptual representations, which emerge in the network solely as a result of (simulated) sensory and motor experience and synaptic plasticity. Secondly, and crucially, we restrict our approach to model exclusively cortical components and physiological functions whose presence in the brain is supported by a large body of neuroscientific evidence. In particular, as opposed to back-propagation learning algorithms, the presence of Hebbian-like synaptic plasticity phenomena in the mammalian cortex stands on very solid experimental grounds^64,65^; we therefore only implement plasticity mechanisms that closely mimic such phenomena^66,67^ and no other learning rules (see below for more details on the structural and functional features modelled here).

The present approach builds upon previous fully brain-constrained computational models simulating the neural mechanisms underlying word acquisition and semantic grounding of language in perception and action^68–74^. These previous studies showed that words and their meaning can be thought of as distributed ensembles of strongly and reciprocally connected cells (known as *cell assembly* circuits, CAs^75,76^). Such CA circuits span several cortical areas, including—but not strictly localised to—multiple semantic hubs (including the ATL), associated modality-preferential cortices, as well as the intermediate areas which may be needed to enable their linkage^70,77^. The presence of such a distributed network of activity in different semantic hubs (ATL, inferior prefrontal cortex, middle/inferior temporal cortex, cortices in the vicinity of the temporo-parietal junction, as well as the inferior parietal and posterior-superior temporal areas of the left hemisphere), and—as predicted by embodied semantics theory—in modality-specific cortices during semantic processes is supported by ample experimental evidence (e.g., see refs.^1,15,78^ for reviews), and should therefore be implemented in the model. While simulating only a subset of brain structures is a necessary simplification pertinent to neurocomputational modelling (see Discussion for a number of other ones), the present choice of areas and connections therein is not only based on this previous research in healthy individuals but also aligns well with data from language/semantic deficits such as svPPA^6,79–82^.

Such previous neuroanatomically and neurobiologically constrained models have been shown to successfully simulate the processes of word acquisition and spontaneous semantic hub emergence^70,72,73^, and even explain well-known patterns of event-related potentials in human neurophysiological data^68,83^. The present study extends this approach to simulate aspects of neurodegeneration associated with semantic dementia. We attempt to explain the neural causes underlying the differential effects that SD is known to exert on word comprehension (which generally declines with an increase in the disease severity) and on word repetition abilities, which tend to be spared^6,32^. In particular, we focus on simulating the effects of progressive SD lesions on the model’s internal distributed representations of two different semantic categories of language items, namely, object- and action-related words.

**Figure 1:**
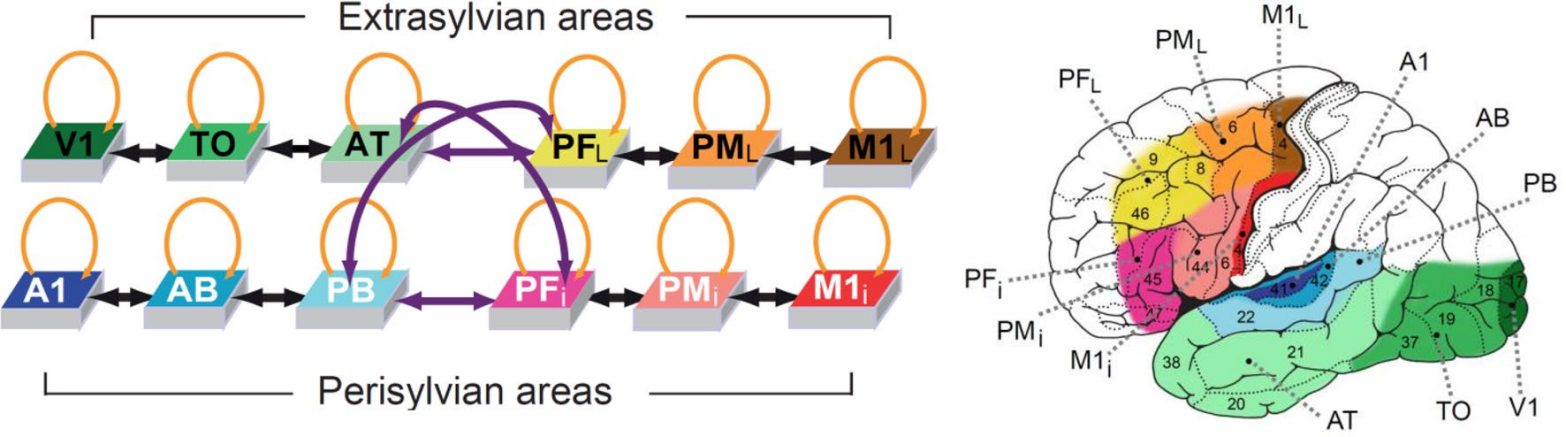
Macrostructure of the model and simulated brain areas. Twelve model areas (left) and corresponding brain areas (right), as coded by colours. There are four ‘zones’ of three different modalities, with three areas in each zone. The ‘auditory’ zone comprises the superior and lateral auditory areas: primary auditory area (A1), auditory belt (AB) and auditory parabelt (PB). The ‘visual’ zone comprises the inferior temporo-occipital areas: primary visual area (V1), temporo-occipital area (TO) and anterior temporal area (AT). The motor cortex is represented by two zones—one corresponds to the articulatory movements, and the other (which we refer to as just the ‘motor’ zone) to nonarticulatory movements. The ‘articulatory’ zone comprises the inferior frontal areas: inferior primary motor area (M1_i_), inferior premotor area (PM_i_) and inferior prefrontal area (PF_i_). The motor zone comprises the superior-lateral frontal areas: lateral primary motor area (M1_L_), lateral premotor area (PM_L_), and dorsolateral prefrontal cortex (PF_L_). We differentiate between the perisylvian cortex (auditory and articulatory areas) and the extrasylvian cortex (visual and motor areas). Black arrows indicate connections between adjacent areas; purple arrows indicate long-distance connections; orange arrows show recurrent, within-area excitatory links (adapted from Ref.^70^, Fig. 1).

**Figure 2:**
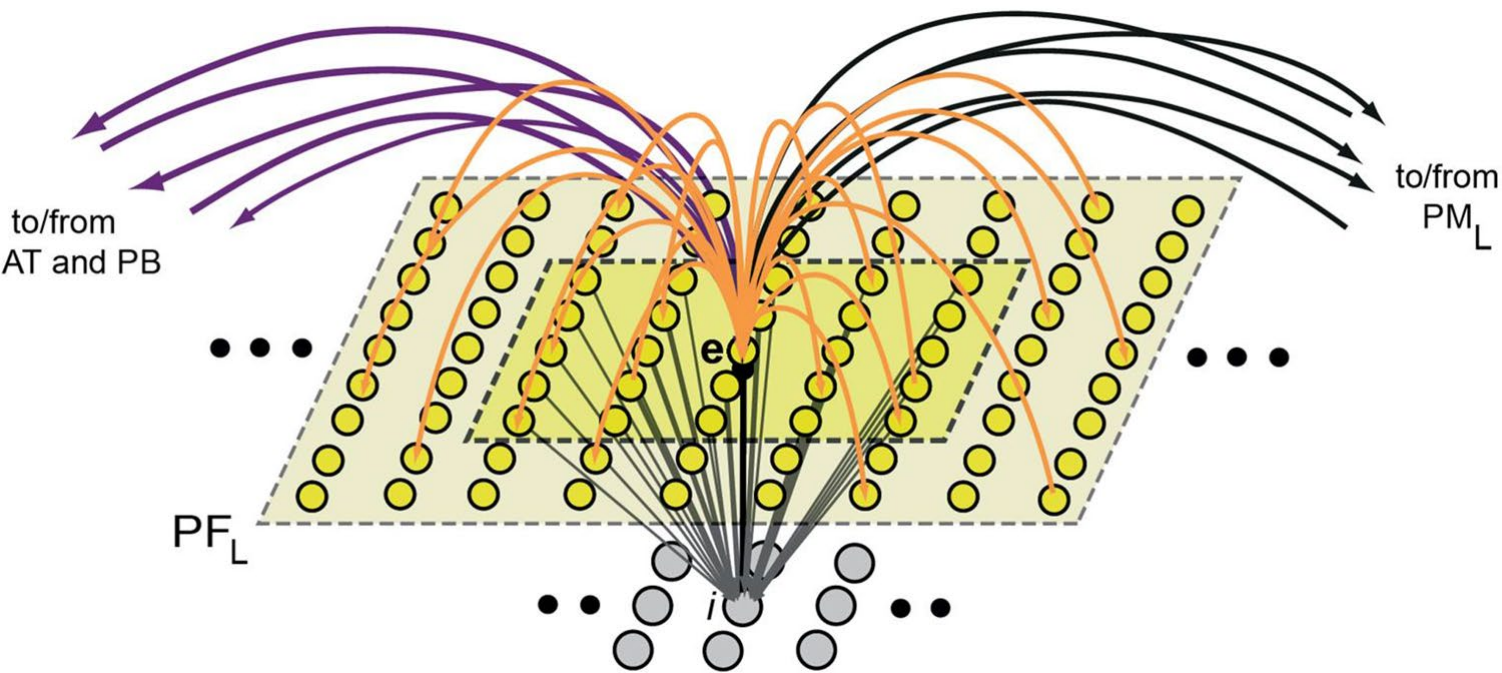
Schematics of microconnectivity of one of the 7500 excitatory neural elements (labelled ‘e’). Within-area excitatory links (in orange) to and from ‘cell’ **e** are random and sparse, and limited to a local (19 × 19) neighborhood (light-yellow shaded area). Lateral inhibition between e and neighboring excitatory elements is realized as follows: the underlying cell ‘i’ inhibits **e**, while its activity depends on the total excitatory input it receives from the 5 × 5 neighborhood around **e** (darker-yellow shaded area); by means of analogous connections (not depicted), **e** can inhibit its neighbors (adapted from Ref.^70^, Fig. 1).

The model (see Figs. 1, 2) closely reflects functional and structural features of the human frontotemporal cortex, and incorporates the following brain constraints (for a recent review of the modelling approach adopted, see Ref.^56^:

*Structure*

1. Twelve cortical areas, known to be implicated in lexico-semantic processing, are modelled; these include modality-preferential sensory and motor ones as well as connector-hub areas which link them;
2. Between-area links in the model (black and purple arrows in Fig. 1) reflect known neuroanatomical links between corresponding brain areas (see Methods for details); recurrent (within-area) connections (not depicted in Fig. 1) are also present, in line with known properties of the cortex^60,84^;
3. Between- and within-area links do not implement *all-to-all* connectivity between cells, but sparse, patchy, and topographic projections, with synaptic links established probabilistically (the probability of two cells being connected decreasing with the distance^60,85,86^) and initialised to weak and random efficacy values.

*Function*

4. Local lateral inhibition^63,87^ and area-specific global regulation mechanisms (referred to as local and global inhibition, respectively)^60,63,76^;
5. Synaptic plasticity (learning), implementing both long-term potentiation and depression (LTP, LTD) mechanisms^67^;
6. Single cells’ neurophysiological dynamics, including sigmoid transformation of membrane potentials into neuronal outputs, as well as adaptation and temporal summation of inputs^88^;
7. Constant presence of uniform uncorrelated white noise (simulating spontaneous baseline neuronal firing) in all neurons of the network during all phases of learning and retrieval^89^.

In addition to improving the neurobiological realism of computational accounts of semantic dementia, we here went one step further and investigated the effects of two distinct types of ATL anomalies reported to be associated with SD—specifically, progressive thinning of cortical grey matter and degradation of incoming/outgoing white-matter fibre connections^5,6,29^. We compared network dynamics resulting from these two simulated SD lesions in terms of levels and loci of impairments of the model's general conceptual processing abilities. Furthermore, in line with both embodied and hub-and-spokes accounts of semantic representations, as configurations of memory traces differ depending on their specific referential aspects, we made use of our architecture to model two different semantic categories—object- and action-related words—in an attempt to investigate the poorly understood subject of category specificity in SD.

We expected that lesions to the model correlate of anterior-temporal semantic hub would lead to progressive degradation of the model’s ability to respond to (“recognise”) learnt word patterns, in line with the known word comprehension deficit in SD. Whereas it is more difficult to make predictions regarding specific degrees of impairment for particular semantic categories or lesion types, in view of the model architecture (see Fig. 1), we expected recognition/integrity of object-related words (grounded in the visual system, more affected by the lesions) to degrade more than that of action-related ones.

## Methods

### Model overview

To address the question of semantic knowledge deterioration during SD, we used a neurobiologically constrained model mimicking relevant cortical areas and their connectivity. The model architecture simulates 12 areas engaged in linguistic and semantic processing in the frontal, temporal, and occipital lobes of the left hemisphere (Fig. 1).

Six of the model’s perisylvian areas—superior temporal, Brodmann areas (BAs) 41, 42, 22, and inferior frontal areas, BAs 44, 45/6—are known to be of vital importance for language comprehension and production^16,90,91^. The other six (hereafter referred to as “extrasylvian”) areas are involved in processing perceptual and motor-related information that pertains to object- and action-related aspects of meaning, respectively. These are areas belonging to the occipital-temporal ‘what’ visual stream (BAs 17, 18, 20, 21), processing information about object identity^92^, and dorsolateral motor and adjacent premotor and prefrontal areas (BAs 4, 6, 8, 46), known to be involved in action planning and execution^93–95^.

Structurally, the model’s between-area connectivity reflects known evidence of neuroanatomical pathways existing between the corresponding brain areas^96,97^ (see next section for details). Each model area consists of two neuronal layers, one of excitatory and one of inhibitory cells, each containing 625 (25 × 25) cells (see Fig. 2). Functionally, model cells are graded-response units, each representing a cluster of excitatory pyramidal cells or inhibitory interneurons. Within-area model structure, single-cell functional features, and synaptic plasticity mechanisms are analogous to those implemented previously (for details, see Ref.^70^) and are reported in the Supplementary Materials.

### Connectivity of the simulated brain areas

The implemented model areas can be thought of as grouped into four sub-systems, each simulating a ‘hierarchy’ of three cortical areas consisting of a primary cortex, the adjacent ‘higher’ secondary, and associative multimodal regions. Neuroanatomical studies show that adjacent cortical areas tend to be reciprocally connected^62,98^. We implemented such connections in each of the four clusters, i.e., within the (1) articulatory (inferior frontal, PF_i_–PM_i_–M1_i_)^98,99^, (2) hand-motor (dorsolateral frontal PF_L_–PM_L_–M1_L_)^94,95,100,101^, (3) auditory (superior and lateral temporal A1–AB–PB)^102–104^, and (4) visual (inferior temporo-occipital V1–TO–AT)^105,106^ systems.

The links connecting non-adjacent model areas (purple arrows in Fig. 1) are also realised in line with evidence on existing long-distance cortico-cortical white matter fibres. In particular, arcuate and uncinate fascicles implement connections between anterior, inferior, and posterior-superior parts of the temporal cortex (areas AT and PB) and inferior prefrontal cortex (area PF_i_)^107–116^. The extreme capsule connects the dorsolateral prefrontal cortex (PF_L_) to anterior-inferior temporal regions (AT)^117–119^ and to the superior temporal cortex (PB)^116,117,120–122^.

### Simulating word acquisition

**Figure 3:**
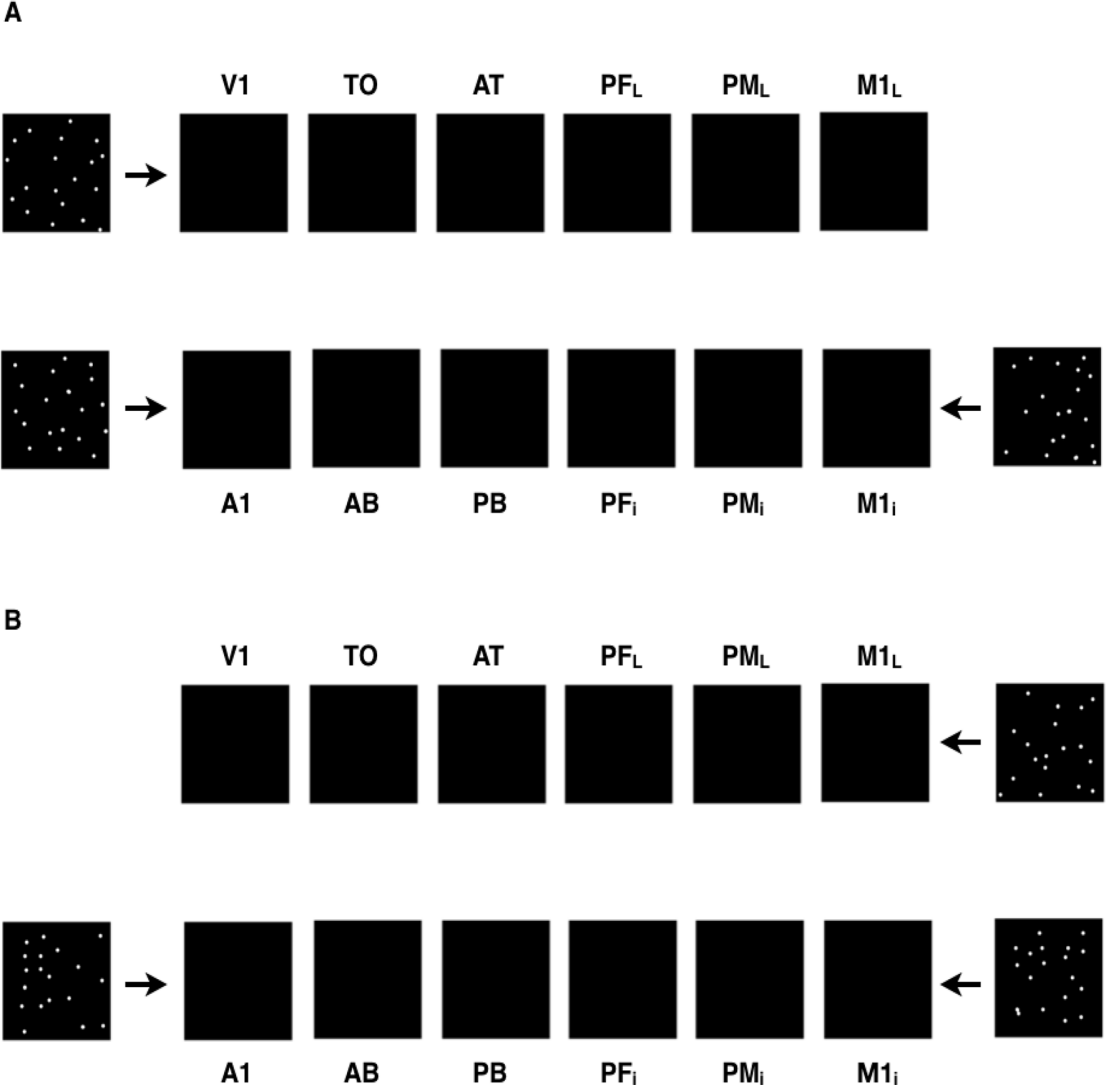
Example of activity patterns presented to the network’s primary areas to simulate word learning. (**A**) The acquisition of object-related words was simulated by providing input patterns simultaneously to the model correlates of the auditory (A1), articulatory (M1_i_) and visual (V1) areas; (**B**) Similarly, the simulation of action-related words acquisition involved presenting concomitant inputs to the auditory (A1), articulatory (M1_i_) and motor (M1_L_) areas. See text for details.

Following a procedure established and consistently used in previous studies^69–73,123^ word-meaning acquisition was simulated through repeated sensorimotor pattern presentations to the primary areas of the network. More specifically, the network was “taught” words having object-related semantics by means of repeatedly co-experiencing auditory, articulatory, and visual patterns provided as inputs to A1, M1_i_, and V1 areas, respectively (Fig. 3A). Similarly, to simulate learning of action-related semantics, input patterns were simultaneously presented to auditory (A1), articulatory (M1_i_), and motor (M1_L_) primary areas (Fig. 3B). Presenting a pattern to one of the above areas involved activating 19 randomly picked cells within the 25 × 25 cells of an area (ca. 3% of the cells).

In line with the standard training procedure, each word pattern was presented over 3000 trials. A trial started with a 16 time-step long word pattern presentation, followed by a period during which no input (interstimulus interval—ISI) was given and the network activity was driven solely by the uniform baseline noise (simulating spontaneous neuronal firing). The next trial started only when the global inhibition of the PF_i_ and PB areas decreased below a specific fixed threshold; this allowed the activity to return to a baseline value, so as to minimise the possibility of one trial affecting the next one. To reflect larger variability of activity in non-involved primary areas (V1 for action-related words and M1_L_ for object-related words), a newly generated random 19-cell noise pattern was provided to them in each trial. Finally, additional (environmental) white noise was also presented as input to the four primary areas during ISIs, aimed at simulating uncorrelated, noisy sensorimotor activity.

The same training process was run in thirteen different network instances (duplicated later, see below), in which all the (between- and within-area) synaptic links connecting single cells were established probabilistically and initialised to random values (see Introduction). Each network instance used twelve different sets of sensorimotor word patterns representing six object- and six action-related words.

**Figure 4:**
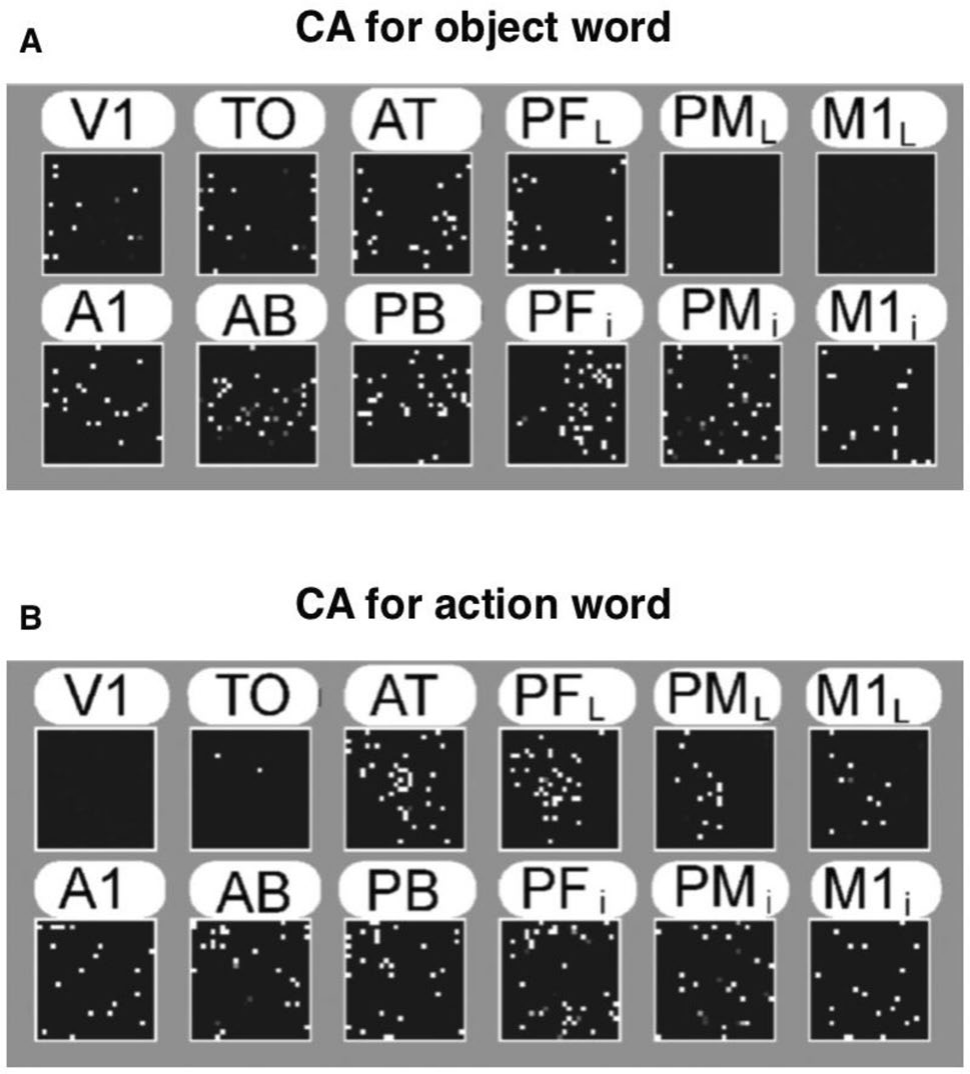
Types of cell assembly circuits emerging in the network as a result of simulated word learning and semantic grounding. (**A**) Representative example of an object-related word CA circuit. (**B**) Representative example of an action-related word CA circuit. Small white dots indicate cells belonging to the CA circuit. CA cells were identified using a standard procedure adopted in previous studies with analogous architectures (see Methods), with CA cells distribution closely replicating previous results70. In particular, note the different distribution of the two semantic types of circuits in the extrasylvian (but not perisylvian) areas of the model. See text for details (adapted from Ref.^70^, Fig. 2).

As previously observed^68,70–73,123–125^, the training procedure described above leads to the formation of stimulus-specific distributed associative circuits (see Fig. 4), or cell assemblies (CAs)^75^, each circuit behaving as a discrete functional unit with two quasi-stable dynamic states (‘on’ and ‘off’) and exhibiting reactivation (or ‘ignition’) in response to the presentation of the stimulus, or part of it. In the present simulations, the emerging CAs (representative examples of which are depicted in Fig. 4) linked model correlates of word forms (circuits in perisylvian areas) to information pertaining to aspects of word meaning (patterns in extrasylvian areas) in a semantic-category specific fashion. More precisely, modelled object-related language circuits extended from perisylvian language areas to the visual (and not the motor) system (including area V1), hence linking up word forms to semantic information in the sensory system; symmetrically, emerging action-related word circuits linked linguistic representations in the perisylvian system to the extrasylvian part of the motor system (including M1_L_), hence connecting word form to aspects of hand-related action meaning. This double dissociation in CA circuit distribution closely replicated previous results^70^.

### Implementation of semantic dementia

Neuronal, anatomical and functional changes during SD can be described as a predominantly left-lateralised pattern of progressive atrophy and hypometabolism both in white and in grey matter^5,6,29^. Grey matter changes are detected in the temporal lobe—superior, middle, inferior temporal gyri, fusiform gyrus, temporal pole (ATL), parahippocampal gyrus—and progress to the basal ganglia and the medial orbitofrontal cortex during the course of the disease (for reviews, see refs^29,126^). White matter changes are detected in regions that are connected or are adjacent to the temporal lobes: left inferior fronto-occipital fasciculus; uncinate fasciculus and inferior longitudinal fasciculus bilaterally^29,126,127^. The most severe and the most robust atrophy in SD patients is detected in the temporal pole, in particular ventral parts of the ATL^41^.

**Figure 5:**
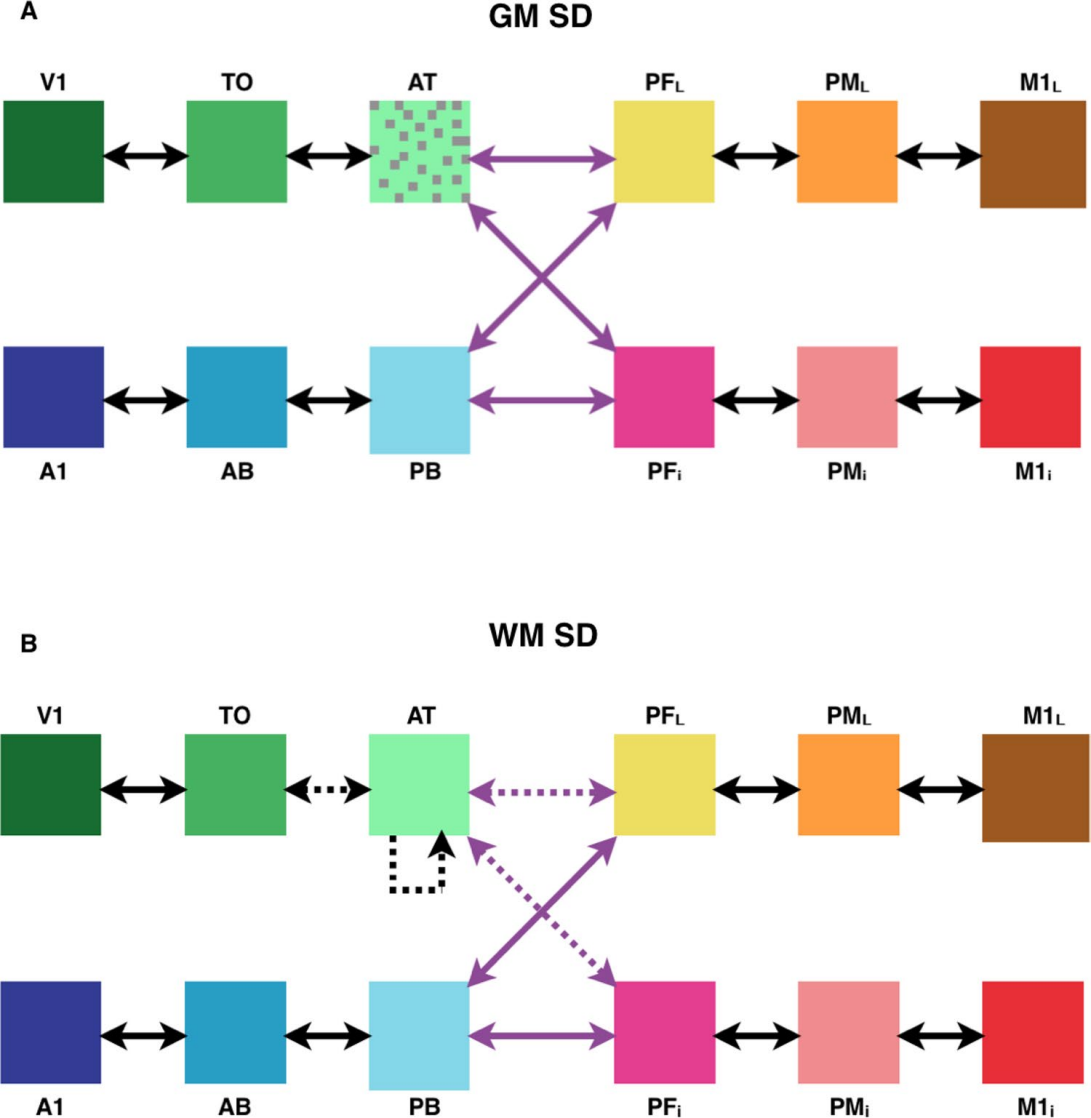
Simulated semantic dementia lesions. Schematic representation indicating the type and location of the model lesions applied to simulate grey matter semantic dementia (GM SD, **A**) and white matter semantic dementia (WM SD, **B**). Dotted area represents inactivation of some portion of e-cells in AT area. Dashed lines represent inactivation of some portion of corresponding links.

In line with that evidence, we applied, in a systematic fashion, both types of degradation to simulate SD—grey matter degradation (GM SD) and white matter degradation (WM SD). To simulate GM SD, we inactivated excitatory cells in the anterior temporal (AT) area (Fig. 5A), while to simulate WM lesions we removed connections to and from those cells (both within- and between-areas; Fig. 5B). To simulate the progressive nature of this disorder^5^, we used four severity levels for each degradation type—0% (no lesion), 30%, 60%, and 90% matter loss.

Thus, two copies of each of the previously-trained network instances were generated, one used to simulate GM SD, the other to simulate WM SD degradation. Each of these 26 networks was tested under the four conditions implementing the different lesion levels. This mimicked a situation in which 26 different SD patients had acquired semantic knowledge of a mini-vocabulary of action- and object-related concepts/words before the onset of the disease.

### Data collection and statistical analysis

Before and after lesion, we recorded the network’s response to the presentation of the auditory-only component (“sound”) of the learnt words. To do this, we presented the A1-pattern of trained words to primary area A1 for two time-steps, while no other input was provided. The activity of each (excitatory) cell in the network was recorded during stimulus presentation and the following 15 simulation time-steps. A cell was considered responsive if its activity during this period reached a given threshold, specified separately for each word pattern (*w*) and model area (A), as follows:

$$\theta \left( {w,A} \right) = \gamma \cdot\mathop {max}\limits_{ e \in A} \left( { \omega \left( {e,t} \right)_{w} } \right)$$

where $\omega$(*e*, *t*)_*w*_ is the (estimated) time-averaged output of a cell $e$ (see Eq. (6) in Supplementary Materials) at time *t*, and γ is a fixed value between 0 and 1 (here γ = 0.50). Simply put, if *m* is the cell maximally responsive to word *w* in area $A$, the threshold $\theta \left( {w,A} \right)$ is 50% of the mean response of this cell to stimulus $w$.

The thresholding function above and the empirically determined value of 0.50 have been consistently used in previous studies to identify CA cells responsive to partial or full CA-circuit stimulation^68,70–73,123–125,128^. In the present context, it is employed as a means to measure the network’s ability to process, or recognise/comprehend, a previously learnt word, from just its auditory (“phonological”) component. Note that, as observed in previous works, partial stimulation of a CA circuit leads to partial reactivation of cells within the circuit, but not to “spurious” responses (i.e., cells not belonging to the stimulated CA circuit are on average not activated above threshold). This is due to the highly stimulus-specific nature of CA circuits^68,124^. Therefore, the total number of responsive cells matches the number of reactivated within-circuit cells very closely. For ease of comparison with results reported in our previous studies, here we chose to adopt the latter as a unit of measure; because of the previously mentioned CA dynamics, this measure implicitly encompasses an assessment of the ability of a distributed CA circuit to fully ‘ignite’ in response to partial (or full) stimulation.

We then used the above measure to compute per-area average numbers of (CA) cells responsive to object- and action-related word “sounds” in each network instance. These were subjected to statistical analysis: to this end, repeated-measures ANOVAs were run for each condition (GM SD and WM SD) with factors ExtraPeri (two levels: extrasylvian and perisylvian areas) and Severity (four levels: 0% [no SD], 30%, 60%, and 90% SD). Further planned (including single-area) and post-hoc comparisons were run using dependent samples t-tests, Bonferroni-corrected for multiple comparisons. To investigate semantic category-specific and lesion type effects, factors Word Type (two levels: object- vs. action-related) and Lesion Type (two levels: grey vs. white matter) were introduced.

The simulations were carried out using Felix, a simulation toolkit for neural networks and dynamical systems^129^, implemented in the C programming language for Linux operating systems, which, besides running the simulations, provides real-time visualisation of the model variables and parameters. The GUI uses XView, a legacy widget toolkit by Sun Microsystems. We ran simulations in a Virtual Machine (VM) using Oracle VM VirtualBox Manager and Ubuntu 14.04. The VM uses a single core and 8 GB of RAM, and a single running simulation requires about 260 MB of RAM. Such a setup can be reliably implemented on any desktop or laptop PC or server as well as the Google Cloud VM service. Partial simulation data are stored during the simulation for further analysis. For the statistical analyses, custom scripts written in the *R* and *Python* programming languages were used: Data curation and display as well as t-tests were performed in *Python* using standard *numpy, pandas, scipy,* and *matplotlib libraries*, whereas ANOVAs were conducted in *R* programming environment using *ez* and *rstatix* libraries.

## Results

**Figure 6:**
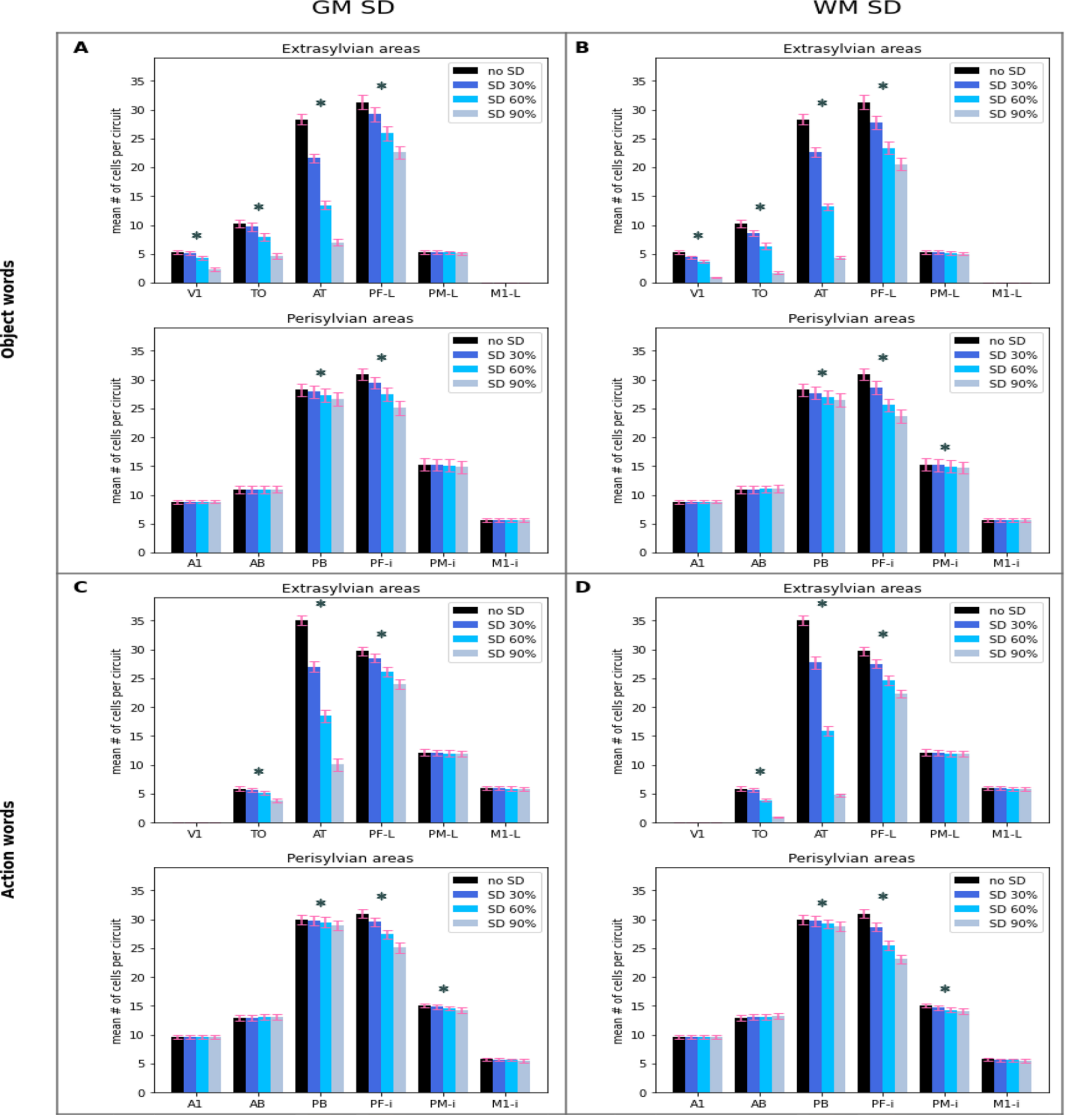
Distributed impact of SD lesions on the network’s auditory word processing abilities. The number of CA cells significantly reactivated by presentation of a learned auditory stimulus (word “sound”) to model correlate of auditory cortex (area A1) is plotted as a function of network area and SD lesion severity. (**A, B**) Responses to object-related words. (**C, D**) Responses to action-related words. (**A, C**) Simulated grey-matter (GM) SD. (**B, D**) Simulated white-matter (WM) SD. Note the reduction in responses particularly visible in the semantic system (extrasylvian areas: top two plots in **A, B** and top two plots in **C, D**). See main text for details. Error bars indicate standard error of the mean. *Difference between no SD and 90% SD cases, *p* < 0.001, t-test.

Figure 6 shows the number of reactivated CA cells in extrasylvian and perisylvian areas of the network in response to A1 stimulation as a function of SD severity, plotted separately for the two semantic categories and lesion types. Statistical analysis using an rmANOVA revealed a significant interaction between factors ExtraPeri and Severity (F_3,36_ = 1166.29, *p* < 0.001), which was driven by differential effects of lesion on extrasylvian and perisylvian areas’ responses, with the former being generally more affected than the latter.

**Figure 7:**
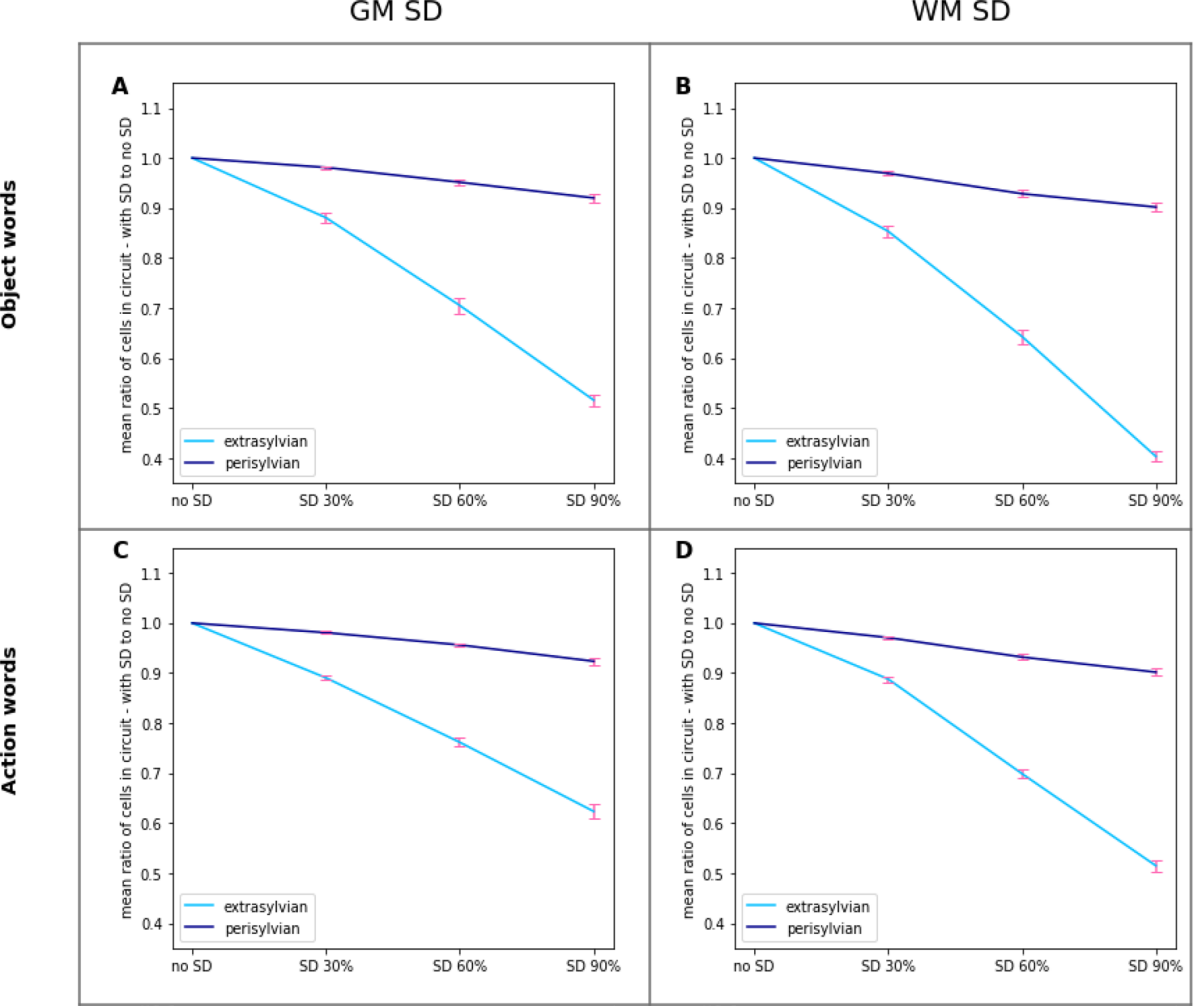
Effects of SD lesions on the network’s word processing abilities. Same data as in Fig. 6 but grouped by extra- (semantic) or peri- (phonological)-sylvian system and plotted as function of lesion severity. Left: grey matter lesions; Right: white matter lesions. Top: object-related words; Bottom: action-related words. The ratios plotted are the percentage of responsive CA cells relative to the total number of CA cells reactivated in the intact network (no-SD condition). Note the significantly stronger decline in responsiveness of the semantic (extrasylvian) system vs. that of the phonological (perisylvian) one as the disease progresses. See the text for details. Error bars indicate the standard error of the mean.

To follow this up and scrutinise differences between extrasylvian and perisylvian responsiveness in individual conditions as a function of SD lesion severity, we ran an rmANOVA with factors ExtraPeri and Severity in each of the four conditions (object- vs. action-related words/GM SD vs. WM SD). This showed a significant interaction between these two factors (F_3,36_ > 307, *p* < 0.001 for all conditions), driven, once again, by larger deterioration of the responses in extrasylvian than in perisylvian areas across all four conditions. The plots in Fig. 7 show extra- and perisylvian-specific numbers of responsive CA cells as a function of SD progression in the four conditions, normalised by the number of responsive CA cells in the “intact” condition (i.e., before SD). Follow-up analysis using t-tests confirmed a significant drop in the number of responsive CA cells between no SD and 90% SD cases in each of the possible semantics/subsystem/damage type combinations (object/action x extra/peri x GM/WM; *t*_12_ > 10.71, *p* < 0.001 for all conditions). On average across conditions, the number of responsive CA cells in extrasylvian areas decreases as SD severity increases by 48.4%; in perisylvian areas this number also declines, with an average drop of 8.7%. This apparent difference in decline rate (which drove the interactions above) was confirmed by additional post hoc comparisons which showed that, in the most severe SD case the number of responsive CA cells in extrasylvian areas was significantly lower than that in perisylvian ones irrespective of the exact semantics/damage type (*t*_12_ > 17.88, *p* < 0.001 in all object/action and GM/WM conditions; this difference was not present before SD). Note, that all results were significant after a Bonferroni correction for multiple comparisons was applied.

To scrutinise the SD influence in a finer-grain detail on the level of individual cortical areas in the model, we performed dependent samples t-tests (Bonferroni-corrected) on the change in the number of responsive CA cells in each of the 12 network areas separately. Results indicate that, in extrasylvian areas, the most severe SD leads to a significant decrease in active CA cells in areas TO, AT, and PF_L_ for both lesion types and semantic categories, and in V1 for object-related words only (see Fig. 6, significant differences are marked with asterisks). CA-cells activity in PM_L_ and M1_L_ was, however, unaffected. In perisylvian areas, SD for both lesion types and semantic categories leads to a significant decline in CA responses in PF_i_, PB and PM_i_ areas, except the object-word GM-SD condition PM_i_ where this decay did not reach full significance.

**Figure 8:**
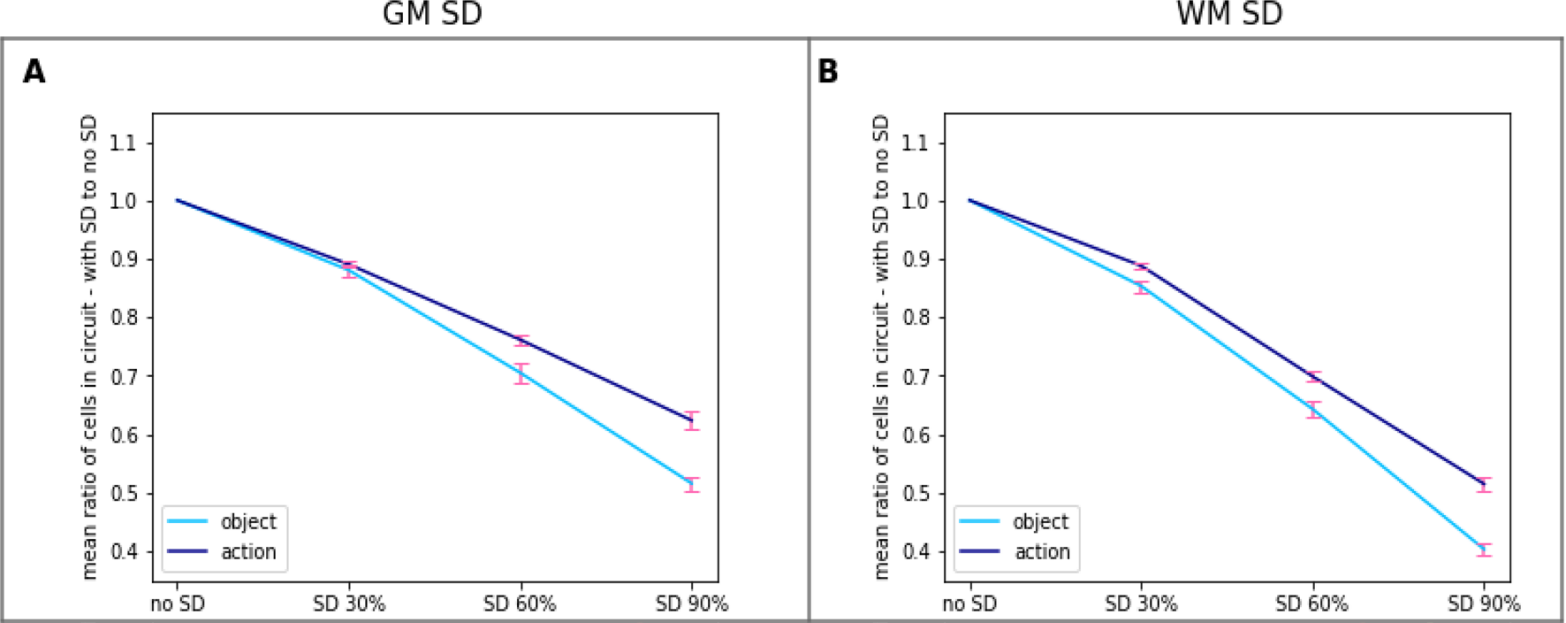
Effects of SD lesions on the network’s word comprehension abilities for action- and object-related words. Same data as in Fig. 7, but from extrasylvian systems only. Left: grey matter lesions; right: white matter lesions. The ratios are computed as the percentage of responsive CA cells relative to the total number of CA cells reactivated in the intact network (no-SD condition). Note the apparently steeper decline in responses to object- than action-related words.

In subsequent analysis we focussed on assessing the effects of SD lesions on category-specific semantics, represented by the extrasylvian (i.e., action- and object-related) areas of the network, which, as the above analysis showed, were more strongly affected by the lesion. Figure 8 plots the percentage of responsive CA cells in this system relative to the total number of CA cells reactivated in the no-SD (intact) condition separately for the two semantic categories (object- and action-related word) in the GM and WM damage conditions, as a function of lesion severity. The plots indicated that the network’s ability to process the meaning of the auditorily presented word declines more for object- than for action-related stimuli. This was confirmed statistically by rmANOVAs ran on the total number of responsive extrasylvian CA cells, which showed an interaction between WordType and Severity factors (GM-SD: F_3,36_ = 7.04, *p* = 0.0012; WM-SD: F_3,36_ = 3.04, *p* = 0.067) as well as main effects of both WordType (GM-SD: F_1,12_ = 11.6, *p* = 0.0052; WM-SD:F_1,12_ = 18.28, *p* = 0.001) and Severity (GM-SD: F_1,12_ = 1252.57, *p* < 0.001; WM-SD:F_3,36_ = 1578.12, *p* < 0.001). Follow-up comparisons showed that, both for GM and WM SD, the network’s overall semantic system’s response to presentation of a known word “sound” decreased significantly more for object- than action-related words (GM SD: 48.3% decline for object words, 37.4% decline for action words, *t*_12_ = 4.61, *p* = 0.001; WM SD: 59.5% decline for object words, 48.5% decline for action words, *t*_12_ = 7.82, *p* < 0.001).

**Figure 9:**
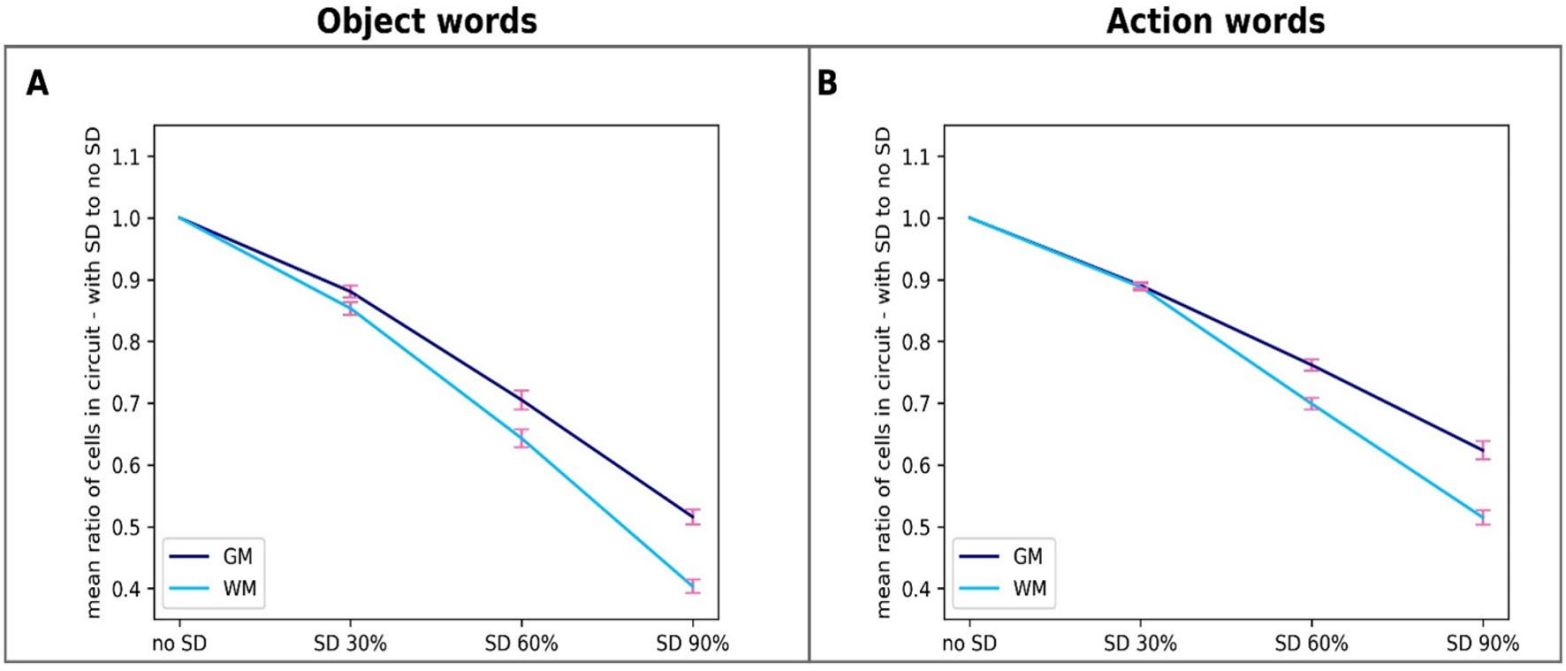
Effects of different types of SD lesions on network’s word comprehension abilities. Same data as in Fig. 7 but restricted to semantic (extrasylvian) areas only and grouped by word type. Left: object-related words; right: action-related words.The ratios plotted are the percentage of responsive CA cells relative to the total number of CA cells reactivated in the intact network (no-SD condition). Notice the apparently steeper decline in responses when WM rather than GM lesions are applied.

The same data are presented in Fig. 9 for the two semantic categories separately. These plots suggest that, both for object- and action-related words, comprehension abilities decline more dramatically in WM SD than in GM SD. To test this observation statistically, we ran rmANOVAs with factors Damage Type (GM vs. WM) and Severity separately for object- and action-related words on the data. In both cases two-way interactions were significant (F_3,36_ > 28.52, *p* < 0.001). Follow-up post-hoc comparisons confirmed that the decline in word comprehension is indeed generally greater for WM SD than for GM SD (object words: 59.5% decline in WM SD vs 48.3% decline in GM SD, *t*_12_ = 8.49, *p* < 0.001; action words: 48.5% decline in WM SD vs. 37.4% decline in GM SD, *t*_12_ = 6.64, *p* < 0.001). Similar effects in perisylvian areas were also significant (object words: *t*_12_ = 5.24, *p* < 0.001; action words: *t*_12_ = 6.41, *p* < 0.001).

## Discussion

We used a neuroanatomically and biologically constrained neural architecture replicating structure and function of relevant frontal, occipital and temporal brain areas implicated in word acquisition, storage and referential semantic grounding to simulate semantic dementia (SD) lesions. The latter were modelled as progressive damage to the network's anterior temporal (AT) area in an effort to explain existing data about language processing impairments in SD patients. After the emergence of distributed cell assemblies (CAs), representing model correlates of cortical circuits for object- and action-related words, we applied two distinct types of progressive tissue degeneration to the model’s AT area that are known to be caused by SD, namely, white- (WM-) and grey-matter (GM) damage, and assessed the network’s word processing and comprehension abilities during presentation of the auditory component of learned words in the different conditions.

As predicted, lesioning the model’s “semantic hub” in the ATL led to a drop in its word recognition performance, in line with the known pattern in SD patients. More interestingly, however, we found diverging effects in different parts of the model. The first such result we report is that, as a consequence of the simulated lesions, the responses in the semantic category-specific (extrasylvian) parts of the model declined more rapidly than those in the core language (perisylvian) counterparts. This difference can be seen as being a direct consequence of the simulated WM and GM lesions to area AT, a crucial “hub” linking the extrasylvian system. A second and perhaps more important result is that the impact of AT lesions on the network’s activity during word processing was manifest not just in AT and surrounding areas (i.e., those directly linked to it—namely, PF_L_, PF_i_, TO, PB), as one might have predicted, but also in areas not directly connected to AT—specifically, in PM_i_ and V1 (see Fig. 6).

The first result is consistent with, and explains, existing data about the decline of language function in clinical populations during onset and progress of semantic dementia. Specifically, the dissociation between word *comprehension* deficits and relatively spared word *repetition* abilities previously observed in SD patients’ data^5,6,32,40,130^ is explained here by the cortically distributed character of word circuits, consisting of cell assemblies (CAs) that link up neurons from both extrasylvian (“semantic”) and perisylvian (“core language”) areas. A large number of experimental studies indicate that the former are involved in conveying information on aspects of word meaning, hence providing a neural substrate for word comprehension processes (see refs.^12,15,131^ for reviews), while the latter mediate the formation and storage of associations between acoustic and articulatory features of syllables and words, i.e., phonological representations engaged in word—and non-word—repetition^16,91^, which, as mentioned, is relatively well-preserved in SD. The reported changes in the network responses after simulated SD lesions are in line with these data: while the perisylvian parts of the distributed CA circuits, needed for successful word repetition, remain relatively unaffected, word comprehension abilities are progressively impaired as SD lesions increase, as a result of the strong reductions in the number of responsive CA cells occurring *across* the extrasylvian system. We submit that these simulated cortical changes can explain, at least in part, the pattern of language impairments observed in SD patients.

That focal lesions in the model’s analogue of ATL lead to degraded responses in areas not directly linked to it is a consequence of the distributed character of CA circuits: because a cell assembly is a set of strongly and reciprocally linked cells, lesioning a specific part of the circuit affects the dynamics of the entire assembly, as the total activity propagating and reverberating in it is diminished^68,77,124^; this is manifest as a pattern of reduced responses distributed across the areas the CA spans, including areas distant from (i.e., not directly linked to) the lesion locus. Thus, the approach used here to evaluate the network’s word comprehension abilities (number of responsive CA cells per area) implicitly relies on the CA circuits’ global dynamics: the decrease in responsive CA cells reported in areas PM_i_ and V1, not directly affected by lesions, must be the result of smaller amounts of activity reverberating within the entire distributed circuit.

Whereas a number of previous attempts at modelling semantic dementia effects on conceptual processing have also been successful in simulating SD, the present endeavour differs from them in some important aspects. Unlike most previous computational accounts^40,44–47,49–51^, in which meaning is implicitly assumed to be processed within a single, amodal module (typically located in the ATL), the present simulations show that, if an a priori locus for meaning is not imposed, conceptual representations naturally emerge as distributed circuits spanning *multiple* hubs (including meaning-related—AT, PF_L_—and language-related—PB, PF_i_—ones), primary sensorimotor (A1, M1_i_, V1, M1_L_) areas, and “in-between” secondary (AB, TO, PM_L_, PM_i_) cortices. The emergence of these simulated correlates of neural word circuits takes place spontaneously and in an unsupervised way, solely as a result of simulated sensorimotor experience and synaptic plasticity mechanisms mimicking well-documented neurophysiological principles of Hebbian associative learning (see section "Simulating word acquisition" above). These principles were implemented in a neural model having structure and features that closely reflect current knowledge of the human cortex’ architectonics; this is another important aspect that distinguishes the present model from previous ones.

As such emerging semantic representations are not localised to a single area, the network’s conceptual processing abilities can only be assessed by evaluating the entire pattern of degraded activity distributed across a system of simulated cortical areas. By doing this we have, in addition to explaining mechanistically some SD-specific patterns, obtained the above novel results regarding the neuroanatomical distribution of functional deficits. Whilst these results are—at this stage—only valid for the present simulations, they constitute a clear prediction that can be tested experimentally in future patient studies: namely, compared to healthy controls, SD patients should exhibit weaker responses during semantic processing not only in ATL and surrounding cortices—directly damaged by the disorder—but also in brain areas far away from the main loci of atrophy. Even more specifically, our results indicate that, when engaging with object-related word comprehension tasks, SD patients’ responses in primary visual cortex (area V1), not directly affected by tissue neurodegeneration, should also be reduced. It is worth noting that no other brain-inspired computational model of SD appears to make this prediction^44,51^. To the best of our knowledge, no compelling neuroimaging data with SD patients are presently available to confirm or refute this prediction, which should be tested in future fMRI or MEG experiments. Such experimental data would therefore allow adjudicating between this and competing computational accounts. That said, some evidence in favour of the above model result does exist: reduced physiological activity in SD patients has been found in a distributed network of modality-specific and association regions linked to the anterior temporal lobe^132–135^ including the left inferior posterior temporal and fusiform gyrus (area TO in the model) and, importantly, the calcarine sulcus (in primary visual cortex).

Another important aspect of the present findings is that they also speak to the issue of category-specific vs. category-general comprehension deficits in SD patients. It is well-known that SD leads to category-general impairments, affecting conceptual knowledge across domains (e.g., tools, animals, etc.) and modalities (visual, action-related, etc.)^1,42,136–138^. Our simulations, however, indicate small but reliable semantic-category differences, with object-related word processing impairments increasing more rapidly than action-related ones as SD progresses. This result follows from the between-area links the model implements (known to exist between homologous brain areas) and the crucial position that area AT itself occupies in the topological structure of the neural architecture. In fact, because of the specific connectivity (refer to Fig. 1), the AT hub is the only “bridge” through which semantic information incoming from the visual system can reach language circuits in perisylvian areas, and drive the formation of object-related word CAs. If the grey matter within—or the white matter bundles that link to—this connector hub are lesioned, such object-related word circuits will be directly affected. Action-related semantic information coming in from the motor system (area M1_L_), on the other hand, can reach the perisylvian word-form areas via a second semantic hub area, PF_L_ (as well as through AT, albeit indirectly); therefore, lesions to the AT hub are expected to affect action-related word circuits to a lesser extent than object-related ones. Interestingly, while there is evidence that SD patients are impaired also in action-related word processing^139^, some studies do report object-naming being more affected than action-naming tasks in such clinical populations^4^. Our simulation results (Fig. 8) suggest that these differences have small magnitude and become significant only at advanced stages of the disease; this could explain, in part, the dearth of positive experimental results. Interestingly, the already mentioned study by Guo and colleagues^132^ found that activity reduction in the calcarine correlated with behavioural deficits in semantic processing during picture-word—but not emotion-word—matching tasks, which is in line with the category specific effects observed here. Unfortunately, other previous studies on word processing in SD patients typically contrasted words from different grammatical categories (i.e., nouns vs. verbs) rather than from different semantic categories^140–144^. Although not unrelated, these lexical-class (verb/noun) and semantic (action/object) contrasts are not identical. Hence, further experimental evidence is required to better assess the validity of the graded semantic-category specific effects predicted by the present model. Given the various confounding factors, this will require, among other things, neuropsychological testing of different semantic categories within the same lexical class of words (nouns).

A further intriguing question raised by our results is why white matter degradation should lead to a more severe word processing decline than proportionally equal loss of grey matter. As discussed in the Introduction, both grey- and white-matter degradation has been found in SD, but their relative input into the functional word comprehension deficit has not been studied sufficiently. Although *functional* connectivity changes in these distributed temporo-frontal networks are well-known from patient studies^80,81^, these are not readily and fully explainable in terms of *structural* connectivity, and the relative contributions of grey- vs. white-matter degradation in the degree of the deficit remain poorly understood. To the best of our knowledge, no patient data are available to confirm the present prediction of more profound effects of white-matter lesions. Whereas future studies using structural MRI-based volumetric and tractographic measures could assess the feasibility of this model prediction in human patient data, in our model this effect may be a direct consequence of the specific character of the distributed CA circuits and of the sparse, random connectivity of the network within which they emerge. Such properties imply that damaging a given portion of the links connecting the CA cells of an area leads—statistically—to a larger impact than damaging the same portion of CA cells themselves. To understand why, consider a simple scenario with just two CA cells. Unlike most other neurocomputational accounts, characterised by full (*all-to-all*) connectivity, within- and between-area projections here are sparse, reflecting a known feature of the mammalian cortex^60,85,86^. Partly because of this^1^, the developing CA circuits are largely disjoint: on average, the percentage of cells shared by any two CA circuits is rather small, empirically estimated to be less than 5% of the total number of cells forming the CA^68,124^. As CAs are mostly disjoint, given two randomly chosen CA cells, the probability of them belonging to the same CA circuit is relatively small (approximately equal to the inverse of the number of distinct CA circuits coexisting in the network, here twelve). Thus, in the vast majority of cases, any two randomly chosen CA cells will belong to distinct CA circuits, and, crucially, will typically not be directly linked. Let us name such arbitrarily chosen cells c_1_ and c_2_. As they belong to different CA circuits, c_1_ and c_2_ will each have strong links to cells within the respective CA. (For a cell to be part of a circuit, it must have at least one incoming link from, and another link projecting to, other cells within that circuit, as, by definition, a circuit must include a closed ʻloop’). Let us call *a* and *b* the two incoming and outgoing links binding cell c_1_ to the CA circuit it belongs to (say, CA #1), and *g* and *d* those linking c_2_ to its respective cell assembly, CA #2 (the rest of this analysis is not affected by the addition of further links to/from cells c_1_, c_2_). It is easy to see that inactivating a given portion of the CA cells considered impacts—on average—a smaller number of CA circuits than removing the same portion of incoming/outgoing links. In fact, assume, for example, that 50% of the CA cells is inactivated; this means that either c_1_ or c_2_ is rendered unresponsive. As c_1_ and c_2_ are not linked, this will always—and thus, on average—affect only *one* of the two CA circuits (and not both). In contrast, removing a randomly chosen half of the four links to/from c_1_, c_2_ will, on average, affect *more* than just one CA circuit. Note, in fact, that there are in total six possible combinations of link pairs:{*a,b*}, {*g,d*}, {*a,g*}, {*a,d*}, {*g,b*}, {*b*,*d*}, of which the last four contain links from *two* distinct CAs (one from CA#1 and one from CA#2) and the first two links that belong to one CA. Thus, if the probability of link damage is uniform over the set of four links, the mean number of CA circuits affected by “cutting” two links (50% the total) at random is 10/6 of a CA (or 1.66 CAs), i.e., more than the mean number of CA circuits affected by inactivation of 50% of the CA cells (which is equal to 1.0 CAs).

While the above simplified scenario may intuitively explain the larger effects of white vs. grey matter lesions on word processing reported here, it remains to be seen whether the same line of reasoning actually applies to the full-scale network simulations. A detailed investigation quantifying the number of synaptic links connecting each CA cell in the network to other CA cells within the circuit it belongs to, and for all network instances, falls outside the scope of this work, but it should be the object of future computational studies. The predicted differences can be also tested by, and guide, future experimental investigations: percentages of model lesions can be directly translated into corresponding portions of brain lesions, and area-specific percentages of grey and white matter losses in SD patients can nowadays be accurately measured using volumetric techniques. Importantly, this would constitute a critical test for the present brain-constrained architecture, as the above novel prediction has not been reported by any of the previous computational models of SD^44,51^.

Whereas the neurobiological accuracy of the present architecture does improve upon previous models of SD, we also acknowledge the limitations of this work, which relies on a number of simplifications. These include both restrictions on model size (e.g., number of areas, number of cells per area, number of ‘words’ taught, number of learning trials, etc.), motivated by the need to keep the simulation time feasible (training of a single network with 12 ‘words’ takes approximately 70 h on a standard Linux server)—as well as on local circuit and cellular complexity (e.g., only one type of excitatory cells, one type of inhibitory cells, one type of synapses, etc.). As reviewed in the Introduction and Methods, the choice/number of areas was based on the established knowledge of the main structures involved in language acquisition and processing (core perisylvian language areas plus sensory and motor ones relevant for action and object semantics) and of neuroanatomical connections between them; the latter are also supported by functional connectivity studies (e.g., MEG activity propagation^145,146^) including, crucially, more recent data obtained in patients with semantic deficits (e.g., resting-state fMRI connectivity^79,80,82^). Any modelling effort by definition entails a simplification of real-life biological systems, and the questions about the mapping and modelling of neurolinguistic systems in terms of complexity, level of detail, granularity, connectivity etc. continue to be debated in the literature^147,148^. In the present case, the validity of the model used, apart from the experimental evidence it is constrained by, is further confirmed by the results that successfully replicate neuropsychological patient data.

In a similar vein, this work involved conceptual simplifications, including, for example, the way in which word meaning is hypothesised to be acquired. It is reasonable to assume that the meaning of at least an initial set of concrete words is grounded in perceptions and actions: children typically acquire the meaning of some words used to refer to familiar objects (like “sun”) in situations involving simultaneous perception of the spoken lexical item and of the referent object itself^149,150^, and a common situation for learning action-related words (such as “run”) involves word usage just before, after, or during execution of the corresponding movement^151^. The training stage of our simulations closely mimics such situations. However, it is known that we learn word meanings also in other ways (in particular, for non-concrete words that lack clear physical referents), as proposed, for example, by syntactic bootstrapping and distributional semantic theories^152–155^. Thus, after an initial core set of concrete concepts has been learned via direct sensorimotor associations, additional meanings (including, e.g., those of abstract words) may be acquired via indirect grounding, i.e., through word-word-correlations mapping^10,156^. While the present simulations do not address these aspects, it is worth highlighting that, in line with such theoretical proposals, recent computational studies carried out with an analogous neural architecture demonstrated that the learning of abstract concepts may be supported by the very same Hebbian plasticity and semantic grounding mechanisms as those used here^71,125^. On a related note, we modelled only the language-dominant left hemisphere (typically most affected in SD/svPPA), whereas fronto-temporal atrophy may often be bilateral. Indeed, FTD patients with predominant right ATL degradation present socio-emotional semantic deficits^157,158^, which have been outside the scope of the present work and remain to be explored/modelled in future investigations.

Another simplifying assumption of this work consists of having simulated each action (and, likewise, visual object) as one static motor (visual) activation pattern. A more realistic modelling could involve time-changing patterns, and/or representing the referential meaning of a word using not just one semantic pattern but several overlapping ones, each representing a sample instance of the same concept. For example, to simulate learning of the concept of “cat”, area V1 could be confronted with different—interleaved—input patterns, each simulating a different instance of the same concept (e.g., cats of different breeds, colours, sizes, etc.). In fact, recent simulations with a spiking architecture similar to the present one have modelled this situation, and reported the emergence of analogous distributed semantic circuits^125^.

To conclude, we here used a physiologically constrained neurocomputational model of frontal, temporal and occipital cortices involved in language acquisition and processing to simulate the effects of semantic dementia on word comprehension abilities. Distributed conceptual circuit structures (cell assemblies for meaningful words) emerged spontaneously in the network—a randomly initialised, sparsely connected and uniform, noisy neural substrate—in a fully unsupervised manner, solely as a result of simulated sensorimotor experience and learning, and without the need to a priori assume a specific brain locus for meaning. Damages to this brain-based model enable us to explain, in terms of functional and structural underpinnings, a range of semantic processing impairments observed in clinical populations—such as the degradation of semantic comprehension abilities under preserved phonological processing—as a result of lesions to anterior-temporal cortex. Furthermore, the present simulation results make novel predictions regarding category-specific effects of semantic dementia on word processing deficits and the relative role of white and grey matter degradation in functional impairments, which can be tested in future patient studies. The fact that such an architecture implements only synaptic plasticity mechanisms and connectivity structure that are known to exist in the human cortex enables the model’s inner workings to speak to corresponding brain processes, suggesting it as a useful tool for guiding future theory-driven investigations of the brain’s semantic system and its deficits.

### Supplementary Information

Supplementary Information.

## Data Availability

Simulation software (including virtual machine and respective code) as well as the data generated and analysed in the present study are available at: https://tinyurl.com/SDneurosim. All software and data are provided *as is*, with no recourse to support; the authors take no responsibility for the use of these by any third parties. Note that no human data have been used in this study, and all data are a result of neurocomputational simulations.
