# Supplementary material for: Breakdown of category-specific word representations in a brain-constrained neurocomputational model of semantic dementia
Source: Sci Rep. 2023 Nov 10;13:19572. doi: 10.1038/s41598-023-41922-8 (PMC10638411; doi:10.1038/s41598-023-41922-8)
Supplement: Supplementary file 1 — Supplementary Information. [file 41598_2023_41922_MOESM1_ESM.pdf]

SUPPLEMENTARY MATERIALS: Model Implementation

Microstructure

Each area consists of two neuronal layers, each of 625 (25x25) cells, one containing excitatory cells and one containing inhibitory ones (in what follows, referred to as e- and i-cells, respectively). To avoid any potential edge effects, layers have a toroidal structure: the top edge is adjacent to the bottom one, and the left edge is adjacent to the right one. In line with Wilson-Cowan models (Wilson & Cowan, 1973), a single pair of e- and i-cell models the average activity of a local population of pyramidal neurons and underlying inhibitory interneurons within one cortical column (grey matter under approximately 0.25 square mm of the cortical surface). Cells are modelled as graded-response neurons (see below).

Each e-cell is restricted to send projections to the 19x19 e-cell neighbourhood within the same area, to topographically corresponding 19x19 e-cell patches in connected areas, and to a 5x5 i-cell patch in the inhibitory layer of the same area (Fig. 2). The probability of a synapse to be created between an e-cell and another cell falls off with their distance (Braitenberg & Schüz, 1998) according to a Gaussian function clipped to 0 outside the relevant neighbourhood. This produces a sparse, patchy and topographic connectivity, as typically found in the mammalian cortex (Amir et al., 1993; Kaas, 1997).

Membrane dynamics

The state of an (excitatory or inhibitory) cell e at time t is uniquely defined by its membrane potential $V(e,t)$, determined by the following equation:

$$\tau \frac{dV(e,t)}{dt} = -V(e,t) + k_1(V_{in}(e,t) + k_2\eta(e,t)) \qquad (1)$$

where $V_{in}(e,t)$ is the sum of all postsynaptic inputs acting upon cell e (see Eq. (2)), $\eta(e,t)$ is a white noise process with uniform distribution over [–0.5, 0.5], τ is the cell's membrane time constant (note that e- and i-cells have different τ, see Table 1),

and k_1 and k_2 are scaling constants. Note that the activity of each e-cell is intrinsically noisy, simulating the spontaneous baseline firing of real neurons (i-cells have k_2=0). The total input to a cell e is defined as:

$$V_{in}(e, t) = (\sum E/IPSPs) - k_G \omega_G(e, t) \qquad (2)$$

where *ΣE/IPSPs* is the sum of all excitatory and inhibitory postsynaptic potentials – I/EPSPs; inhibitory synapses are given a negative sign – acting upon neural cluster (cell) e at time t, $\omega_G(e, t)$ is the global (or area-specific) inhibition (see Eq. (3)) and k_G is a scaling constant. Note that each e-cell gets exactly one IPSP from its twin i-cell (see Fig. 2).

The global inhibition mechanism is an area-specific inhibitory loop that prevents overall network activity from falling into non-physiological states (Braitenberg & Schüz, 1998). (Note that k_G =0 for i-cells: global inhibition acts only on e-cells for simplicity). Specifically, for each model area A, the global inhibition $\omega_G(e, t)$ is defined by:

$$\tau_G \frac{d\omega_G(e,t)}{dt} = - \omega_G(e, t) + \sum_{e \in A} O(e, t) \qquad (3)$$

where $\sum_{e \in A} O(e, t)$ is the sum of all e-cell outputs within area A (see Eq. (4)) and τ_G is the global inhibitory response time constant.

All cells produce a graded response representing the average firing rate of the neural cluster; in particular, the output (transformation function) of an e-cell e at time t is defined as:

$$O(e,t) = \begin{cases} 0 & if\ V(e,t) \le \varphi(e,t) \\ V(e,t) - \varphi(e,t) & if\ 0 < (V(e,t) - \varphi(e,t)) \le 1 \\ 1 & otherwise \end{cases} \qquad (4)$$

Eq. (4) above is a piecewise-linear sigmoid function of the e-cell's membrane potential $V(e, t)$, clipped into the range [0, 1] and with slope 1 between the lower and upper thresholds $\varphi(e, t)$ and $\varphi(e, t) + 1$. The output $O(i, t)$ of an i-cell i is 0 if $V(i, t) < 0$, and $V(i, t)$ otherwise (i.e., unlike e-cells, i-cells do not saturate reflecting that real interneurons show little firing rate adaptation).

The threshold $\varphi(e, t)$ of an e-cell is not constant but depends on the cell's recent activity, so that the more active the cell, the higher the threshold (see Eq. (5)). This implements a simple form of homeostatic adaptation (Matthews, 2001):

$$\varphi(e, t) = \alpha\, \omega(e, t) \qquad (5)$$

where $\omega(e, t)$ is the estimated time-average of cell *e*'s recent output (see Eq. (6)) and α is a scaling constant (adaptation strength). The estimated time-average $\omega(e, t)$ of a cell's output is computed by integrating the following differential equation (Eq. (6)) with time constant τ_A, assuming $\omega(e, t)=0$ at time $t=0$:

$$\tau_A \frac{d\omega(e,t)}{dt} = -\ \omega(e, t) + O(e, t) \qquad (6)$$

Following previous simulations for computing the cell assembly circuits (see Methods) we used area- and stimulus-specific thresholds $\theta(w, A)$ based on all cells' estimated time averages $\omega(e, t)$ taken at time $t = 16$ post-stimulus onset.

Synaptic weight dynamics

Initially, all established synaptic links between two e-cells are assigned to random values uniformly sampled from [0, 0.1]. At each simulation time-step, the weights between e-cells are allowed to change, according to the following Hebbian learning rule:

$$w_{t+1}(x, y) = \begin{cases} w_t(x, y) + \Delta w & \text{if } O(x, t) \geq \theta_{pre} \text{ and } V(y, t) \geq \theta_+ & (LTP) \\ w_t(x, y) - \Delta w & \text{if } O(x, t) \geq \theta_{pre} \text{ and } \theta_- \leq V(y, t) < \theta_+ & (LTD) \\ w_t(x, y) - \Delta w & \text{if } O(x, t) < \theta_{pre} \text{ and } V(y, t) \geq \theta_+ & (LTD) \\ w_t(x, y) & \text{otherwise} \end{cases} \qquad (7)$$

where $w_t(x, y)$ represents the weight (synaptic efficacy) of the link from cell x to cell y at time t, and Δw is a small positive value (Δw <<1) indicating the weight change a link may undergo at each simulation time-step.

Following Artola, Bröcher and Singer (Artola et al., 1990; Artola & Singer, 1993), the above weight-update rule – known as the "GWP" rule – accurately replicates well-documented synaptic plasticity phenomena of long-term potentiation (LTP) and depression (LTD), hence covering both Hebbian and "anti-Hebbian" phenomena. Specifically, Eq. (6) implements voltage-dependent synaptic plasticity with one *fixed* pre-synaptic threshold θ_{pre} (representing the minimum level of presynaptic activity required at a synapse for any weight change – LTP or LTD – to occur) and the current postsynaptic potential $V(y, t)$ determining the "sign" of the change, based on two *fixed* post-synaptic thresholds θ_- , θ_+ (see (Garagnani et al., 2009) for a discussion on the neurobiological realism of the GWP rule).

Table 1. *Model parameters*

$\tau_e = 2.5$	e-cells membrane potential time constant (Eq. (1))
$\tau_i = 5$	i-cells membrane potential time constant (Eq. (1))
$\tau_G = 12$	global inhibition time constant (Eq. (3))
$\tau_A = 10$	e-cells estimated time-averaged activity time constant (Eq. (6))
$k_1 = 0.01$	scaling constant (Eq. (1))
$\eta \sim U[-0.5, 0.5]$	noise distribution (Eq. (1))
$k_2 = 100\sqrt{3}$	noise amplitude (Eq. (1))
$k_G = 95$	scaling constant (Eq. (2))
$\alpha = 0.01$	adaptation strength (Eq. (5))
$\Delta w = 0.0008$	learning rate (Eq. (7))

$\theta_{pre} = 0.05$	presynaptic firing- rate threshold for LTP / LTD (Eq. (7))
$\theta_{-} = 0.15$	postsynaptic potential threshold for (homosynaptic) LTD (Eq. (7))
$\theta_{+} = 0.15$	postsynaptic potential threshold for (heterosynaptic) LTD (Eq. (7))
